# Recognition and Detection of Wide Field Bionic Compound Eye Target Based on Cloud Service Network

**DOI:** 10.3389/fbioe.2022.865130

**Published:** 2022-04-04

**Authors:** Yibo Han, Xia Li, XiaoCui Li, Zhangbing Zhou, Jinshuo Li

**Affiliations:** Nanyang Institute of Big Data Research, Nanyang Institute of Technology, Nanyang, China

**Keywords:** cloud service network, wide-field bionic compound eye, image localization, target recognition detection, energy symmetry

## Abstract

In this paper, a multidisciplinary cross-fusion of bionics, robotics, computer vision, and cloud service networks was used as a research platform to study wide-field bionic compound eye target recognition and detection from multiple perspectives. The current research status of wide-field bionic compound-eye target recognition and detection was analyzed, and improvement directions were proposed. The surface microlens array arrangement was designed, and the spaced surface bionic compound eye design principle cloud service network model was established for the adopted spaced-type circumferential hierarchical microlens array arrangement. In order to realize the target localization of the compound eye system, the content of each step of the localization scheme was discussed in detail. The distribution of virtual spherical targets was designed by using the subdivision of the positive icosahedron to ensure the uniformity of the targets. The spot image was pre-processed to achieve spot segmentation. The energy symmetry-based spot center localization algorithm was explored and its localization effect was verified. A suitable spatial interpolation method was selected to establish the mapping relationship between target angle and spot coordinates. An experimental platform of wide-field bionic compound eye target recognition and detection system was acquired. A super-resolution reconstruction algorithm combining pixel rearrangement and an improved iterative inverse projection method was used for image processing. The model was trained and evaluated in terms of detection accuracy, leakage rate, time overhead, and other evaluation indexes, and the test results showed that the cloud service network-based wide-field bionic compound eye target recognition and detection performs well in terms of detection accuracy and leakage rate. Compared with the traditional algorithm, the correct rate of the algorithm was increased by 21.72%. Through the research of this paper, the wide-field bionic compound eye target recognition and detection and cloud service network were organically provide more technical support for the design of wide-field bionic compound eye target recognition and detection system.

## 1 Introduction

In the process of target recognition, the target object to be detected often undergoes interference such as variable speed, deformation and occlusion, when the recognition algorithm using image feature matching will be greatly affected in terms of computational accuracy, and it is almost impossible to distinguish and recognize the target object that is difficult to define features by such methods (Zhiwen Yang et al., 2021). The integration of bionic compound eye technology and target recognition technology is an important direction to truly realize the multi-environment generalization and artificial intelligence of machine vision platforms (Bai et al., 2022; Jiang et al., 2019a). A machine vision detection system that combines the above functions will have stronger detection and recognition capabilities, greatly improving target detection efficiency and multi-environment adaptation capabilities. Traditional computer technology and network technology, so the use of cloud service network has higher reliability compared to traditional computers, improving the efficiency and security of network services. Of course, the cloud service network also has the characteristics of versatility and scalability, its application areas are more extensive, not only for a specific field, and the same cloud service network can support the operation of different applications (Wu et al., 2022; Ying Liu. et al., 2022b); at the same time, according to the size of the user, the scale of cloud service network can be dynamically scaled to meet the needs of users on-demand, which also ensures the distribution of network resources and services on-demand. Unlike traditional imaging systems, the insect compound eye has multiple imaging channels and can fuse images captured by multiple imaging channels to achieve a wide field of view imaging and real-time target tracking (Dong et al., 2020; Dupont et al., 2021). In addition, the insect compound eye has the advantages of small size and lightweight, making it one of the ideal visual localization systems. Inspired by the insect compound eye, researchers have designed various artificial bionic compound eyes using microlens arrays as sub-eyes, which have the characteristics of small size, wide field of view and high sensitivity. The mechanism of biological vision at a higher level is not yet fully understood, and some mechanisms are still controversial, and the accuracy of the established models needs to be further explored (Grischke et al., 2020; Hao et al., 2021). Because of the virtualization, scalability, flexibility and efficiency of cloud computing, it is more flexible to perform operations on the cloud platform. Before the operation is carried out, it is necessary to take efficient safety measures to build a safe and reliable operating environment, to ensure the safety of the system, and ensure that the operation is completed on time.

The conventional imaging system can only capture the spatial information in the target field of view, but the bionic compound eye system can also capture the directional information of the light in the field of view (Yun et al., 2022), and the integrated use of spatial and directional information of light enables the use of the system for light field imaging; on the other hand, the bionic compound eye imaging system can image the object space through multiple sub-eyes, and thus has a large field of view, which can be achieved with small system size (Harrer et al., 2019). On the other hand, the bionic compound eye imaging system has a large field of view through multiple sub-eyes to image object space, so it can achieve 180° or even 360° imaging range with a small system size; moreover, it can achieve rapid detection and localization of moving targets based on the high sensitivity of the compound eye to respond to moving targets. According to the characteristics of the biological compound eye sensitive to polarized light, it can design polarized light-sensitive bionic compound eye system. The own characteristics of the bionic compound eye imaging system make its numerous applications in common target location identification, fast-tracking, UAV obstacle avoidance, navigation, range measurement, speed measurement and other fields. Since the structure of the bionic compound eye imaging system tends to detect the shape features of the target rather than local details in use, its low resolution limits the full play of its advantages (Huang et al., 2021). Even though the imaging capability of a single sub-eye is weak, the compound eye has a wide field of view and has multiple channels of imaging information, and this feature makes compound eye imaging a hot research topic. In this paper, we plan to design a fast target detection and tracking algorithm based on the idea of compound eye vision by analyzing the mechanism of the insect compound eye vision system (Jiang et al., 2019b). The algorithm is different from most of the existing motion detection and tracking algorithms in terms of idea and method and is expected to get a special effect on low spatial resolution video analysis. In addition, it can be used not only for booth effect evaluation but also in many other applications, such as intelligent surveillance and human-computer interaction. In these applications, fast target discovery and tracking are fundamental to the implementation of the whole system, while reducing power consumption is also a common requirement for them (Karakaya et al., 2020; Kas et al., 2021). The algorithm can be 0easily combined with other methods to synergistically meet the needs of various applications (Lin et al., 2020; Ying Liu et al., 2022a; Xin Liu et al., 2022).

In this paper, a wide-field bionic compound-eye target recognition and detection network architecture based on a cloud service network is proposed to effectively improve the network performance of cloud service networks and deploy the designed trust assessment algorithm (Liu et al., 2020). With the support of cloud computing services, a three-layer cloud network architecture is constructed, including a central cloud layer, a roadside base station cloud layer and a target cloud layer, where different cloud layers have different functional roles and each cloud layer is interconnected and cooperative with each other. Chapter 1 is the introduction section, which introduces the related research background and significance of this topic, and also analyzes the importance of cloud service network performance and information security, outlines the main content of this thesis, and then introduces the organization of this thesis. Chapter 2 is about our research. In this part, the related research works of this thesis are introduced. Firstly, the system architecture and characteristics of cloud service networks are studied, and the development opportunities and the current status of domestic and international research on wide-field bionic compound eye target recognition and detection for cloud service networks are outlined to lay the foundation for the proposed scheme of the thesis. Chapter 3 presents the research of wide-field bionic compound eye target recognition and detection based on the cloud service network. Firstly, a wide field bionic compound eye cloud service network model is constructed. Then, we further refine the relevant wide-field bionic-eye target recognition and detection algorithms based on the direct and indirect connections and regional connections of the nodes in the cloud service network. Finally, the wide-field bionic compound-eye target recognition and detection of the cloud service network studied in this paper is designed as a system to fully apply the research algorithms in this paper. Chapter 4 is the result analysis part. To verify the feasibility and practicality of the bionic compound eye target detection system in the actual application environment, the system is tested using the real target detection environment to verify that the designed system meets the design index and can achieve accurate and fast recognition of multi-scale targets in a large observation range. Chapter 5 is the conclusion section. It summarizes all the contents of this paper and gives an outlook on the future research contents.

## 2 Related Work

The bionic compound eye has a large detection range, high detection flexibility, small size, and lightweight, and has great application value and research potential in military missions, industrial production, aerospace, measurement, mapping, etc. Many scholars at home and abroad have conducted corresponding research on bionic compound eye systems (Mackenzie and Munster, 2019). They used photolithography to fabricate a cloud service network bionic compound eye system. The complex-eye system uses transparent glass as the substrate and uses photolithography to process light-transmitting holes and microlens arrays on its front and back sides, respectively. After the image light signal passes -3,354 through the microlens, it will directly pass through the corresponding Transmissive aperture under the lens and finish imaging on the photoelectric receiver. They reject the conventional view that the sweeping motion and smooth tracking motion are controlled by separate mechanisms (Ren et al., 2019; Romano et al., 2019). The traditional view is that the position error drives the sweeping motion and the velocity error drives the smooth tracking motion. However, in the paper, the author showed that sweeping motion and smooth tracking motion were two outputs controlled by a single sensorimotor mechanism, which shared the same input signals, i.e., position error and motion velocity error, and their outputs collaborate for the common purpose of optimally tracking the target (Shao et al., 2020; Fuqin Sun et al., 2021). This algorithm generates the best countermeasures that can be used against the opponent based on the opponent’s movements obtained from the robot’s high-speed vision system. The problem of poor edge-lens imaging quality is further solved by introducing curved field mirrors into the artificial compound-eye imaging structure and obtaining a wide field of view. The three-layer curved compound eye structure has a field of view angle of 88°. Although its structure is bulky, its edge-field imaging quality is significantly improved (Zhongda Sun et al., 2021). The bionic compound eye system designed and implemented concerning the natural structural characteristics of insect compound eyes has the advantages of the large field of view, small size, and low power consumption compared with the multi-eye vision system composed of multiple cameras; The channels share a large area array CMOS sensor, which greatly reduces the amount of data and is more practical. It can be used in target detection and surface measurement (Talaviya et al., 2020).

They studied the compound eye through the cloud service network, and proposed to use the cloud service network refraction imaging principle and adopt the high sub-free-surface instead of the microlens array to achieve the purpose of high imaging quality, and adopt the hexagon as the single sub-eye lens structure to eliminate the dead angle of imaging, and adopt the overall surface structure to widen the field of view. To achieve the goal of small volume imaging, they studied the cloud service network design of a new concave and planar overlapping compound eye system, in which each channel contains three aspherical lenses and each channel images all fields of view, improving the imaging resolution and sensitivity (Wang and Qi, 2021). They proposed a bionic compound eye imaging cloud service network architecture and designed a curved bionic compound eye cloud service network bionic compound eye system with a field of view of 60° and a seven-channel multilayer curved compound eye cloud service network bionic compound eye system with a field of view of 180° (Wang et al., 2020). Six subocular heads at the edge form a subocular lens array around a central subocular, containing a total of seven subocular heads for the artificial compound eye system, and the imaging effect of the system is explored using indoor experiments, and the image processing method is used to stitch the acquired images (Wang et al., 2019). The rapid improvement of micro and nano processing and flexible electronics has led to the research on the fabrication of curved compound eyes, whose wide field of view and lightweight have led experts and scholars to increase their research efforts on curved compound eye systems (Wei et al., 2021). However, because the curved image cannot be received by the flat image detector, it is still difficult to design and process the curved compound eye imaging system, which limits the further development of the curved compound eye system (Wu et al., 2019; Xiang et al., 2019).

After the above analysis and research on the development status and application prospect of the bionic compound eye system, it is known that it has great research value in detection and identification, imaging, etc. Its application prospect can touch the military field of detection and reconnaissance, and also can be involved in the application of civil engineering (Cheng Yang et al., 2021). This paper establish the principle model of the wide field of view bionic compound eye target recognition and detection system design for cloud service network. It uses the model to complete the design of wide field of view curved bionic compound eye cloud service network bionic compound eye system, and initially use super-resolution reconstruction algorithm to realize the wide field of view high-resolution imaging as the main research objective (Yu et al., 2020; Sun et al., 2022). Considering that the quality defects of the side-view video itself are difficult to overcome by purely algorithmic methods, this design adds a top-view camera (the viewpoint need not be completely downward, it can be the viewpoint above the side), which is mainly responsible for pedestrian detection and tracking and counting out the total number of people. Since the occlusion phenomenon is not obvious in the top view video and the background changes are small, the location and size information of the moving target can be obtained more reliably, and this information can provide the side view camera with a reliable search location and spatial range of the face target, significantly reducing the face recognition frequency and face pattern search space. In this way, by fusing the video analysis results of top-view and side-view cameras to complement each other’s strengths and weaknesses, the above deficiencies can be largely compensated.

## 3 Research on Wide-field Bionic Compound Eye Target Recognition and Detection Based on Cloud Service Network

### 3.1 Wide Field Bionic Compound Eye Cloud Service Network Model Construction

**FIGURE 1:**
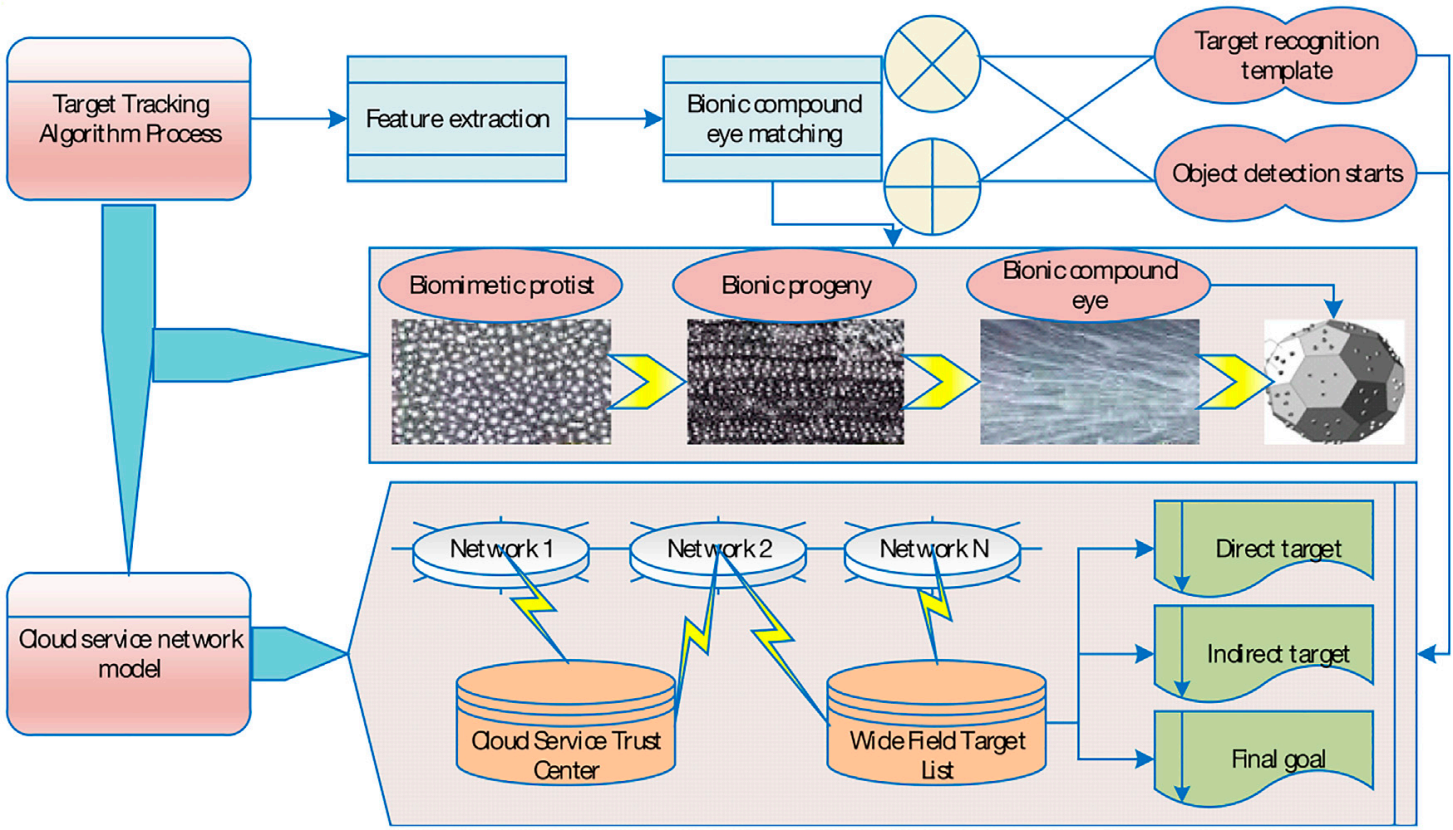
General flow chart of the tracking algorithm.

In the process of target tracking, since the target’s pose, scale and illumination changes can affect the reliability of template matching, a template update strategy needs to be designed to correct the template promptly and improve the tracking accuracy. The basic idea of the template matching tracking algorithm is to use the feature information of the target image to build a template, and then match the established template with the image to be tracked to search for the target location. In this paper, the basic idea of pedestrian tracking algorithm based on template matching, firstly, local features are extracted from the detected pedestrian target image, the search area is determined and so matched, the highest matching similarity is calculated, and if the similarity is greater than the threshold value, the target template is updated; otherwise, the pedestrian target is detected again. The flow chart is shown in Figure 1 (Zhu et al., 2020). The imaging process of both the eye and the camera consists of projecting the three-dimensional world onto a two-bit plane, thus losing the depth information such that we cannot recognize its distance and actual size from the image (Sun et al., 2020). In the template matching algorithm, the size of the template image has a great influence on the accuracy and speed of matching. When the size is small, it is easy to cause matching errors. When the size is large, the assumption that each pixel in the template block performs translational motion may not be satisfied, thereby reducing the matching accuracy. Therefore, when selecting the template image size, it is necessary to determine the most appropriate size according to the actual effect.

The cloud service network first takes the whole image as the network input and uses the set image segmentation grid to slice the image into s∗s small image blocks, which will be passed into the convolutional neural network as the input. The convolutional layer takes the input data and performs convolutional computation by the convolutional kernel to extract the feature values of the input data, before giving limits to the range of output values by an activation function. In the cloud service network, the activation function used in the convolutional layer is the linear activation function, which is defined as

$$Q\left(m\right)=\langle \begin{array}{c} \frac{m}{10},m\leq 0\\ m,m>0 \end{array}$$ (1)

The pooling method used in the cloud service network is maximum pooling, which selects the largest value in each segmented region as the feature value of that region. Usually, the pooling block size is selected as 3∗3 kernels, if the image is large a 5*5 pooling kernel can also be taken. Two fully connected layers are used in the cloud service network, while the original fully connected layer is deleted in the cloud service network and the convolutional layer is used for downsampling. The final output layer classifies the one-dimensional feature vector input from the fully connected layer and outputs the classification number of the target object to determine the type of the target object in the image (Zhu et al., 2021).

The mean-squared sum error is used as the loss function to optimize the model parameters, and the loss function is shown in Eq. 2. Where ZE is the coordinate error between detection data and calibration data, IE is the IoU error, and CE is the classification error. Since the impact of position-related errors (coordinates, IoU) and classification errors on the network loss function is different, the network adds corrections to correct the coordinate errors when calculating the loss function. When calculating the IoU error, both the grid containing objects and the grid without objects are calculated, and the effect of the IoU error on the loss function is also different.

$$L\left(m\right)=\sum_{k=0}^{Q^{2}}\left(CE+ZE+IE\right)$$ (2)

When we choose the learning rate, we first choose an estimate of the learning rate threshold when the cost function on the training data starts decreasing immediately rather than oscillating or increasing. This estimate does not need to be too precise, but can first be roughly estimated in magnitude. If the value of the cost function starts to jitter or even increase at a learning rate of 0.01, then gradually try learning rates of 0.001, 0.0001, et al. Until you find the learning rate at which the cost function decreases after the start of training. By following this approach, the estimation of the magnitude of the threshold of the learning rate can be quickly grasped, so that the optimal learning rate can be optimally estimated.

Since the parameters of the sub-eye lenses are determined by considering the manufacturing process requirements of the microlens array, the selected solution for the preparation of the microlens array in this paper is the injection molding process, and the contact angle of the mold made by this process is larger than that formed by the photoresist hot melt method, which can reach 30°. Since the radius of curvature R(m) for each sub-eye lens has been determined during the design of curvature focal length, the crown height H(m) and aperture D(m) for each sub-eye lens can be obtained from the geometric relationship, as shown in .

$$\{\begin{array}{c} H\left(m\right)=R\left(m\right)*\left(1-\sin\phi \right)\\ D\left(m\right)=2*\lambda *R\left(m\right)*\cos\phi \end{array}$$ (3)

The known radii of curvature of the sub-eye lenses are substituted into Eq. 3 to obtain the crown height and aperture of the sub-eye lenses, the parameters of the sub-eye lenses are shown in Table 1. To improve the imaging quality of the sub-eye lens, RMS is selected for optimization. The currently set optimization function is the system default, not the best optimization. It will only make the speckle size as small as possible, so more optimization functions need to be set.

**TABLE 1:** Table of dimensional parameters of sub-eye lenses of each stage (mm).

Parameter	Sub-eye Series					
	0	1	2	3	4	5
D(m)	1	0.98	0.94	0.91	0.86	0.82
H(m)	0.15	0.14	0.11	0.09	0.07	0.06

Combined with the specific application context, the camera used by the bionic eye is a digital camera, and the image plane is a W × H grid of photosensitive cells, which is also called a pixel. It corresponds directly to the image pixels, so it is more convenient to express the image plane coordinates in pixel coordinates. The pixel coordinate is a two-dimensional non-negative vector (W, H), and by convention, the far point of the image coordinate system is generally in the upper left corner of the image. So the pixel coordinates are directly related to the image plane coordinates, as shown in Eq. 4. Where P(w) and P(h) are the width and height of each pixel, respectively, and (W_0_, H_0_) is the principal point coordinate principal point is the intersection of the image plane with the optical axis.

$$\{\begin{array}{c} W=\sum_{i=1}^{n}\frac{m}{P\left(w_{\text{i}}\right)}+W_{0}\\ H=\sum_{i=1}^{n}\frac{m}{P\left(h_{i}\right)}+H_{0} \end{array}$$ (4)

Next, consider the chi-squared coordinates O(m) of point m under the camera coordinate system. Since we often use the world coordinate system, we need to establish the conversion relationship between the chi-squared coordinates M^W^ of point m under the world coordinate system and the chi-squared coordinates under the camera coordinate system O(m). According to the basics of coordinate conversion, it is shown in.

$$O\left(m\right)=T{\left(m\right)}^{W}*M^{W}$$ (5)

In actual products, due to the uneven processing conditions, it is generally difficult to obtain an accurate frontal internal parameter matrix just by theoretical calculation. In most cases, it still needs to be calibrated by a calibration plate to obtain it. As for the external parameters, this problem exists, but on the bionic eye platform, the external parameters can be calculated in real-time by the pose matrix. In this paper, the actual internal parameters of the camera are obtained by calibration, while the external parameters are obtained by the pose matrix. In the case of the bionic eye stage, which is a nine-degree-of-freedom binocular vision stage, the mathematical model is more complex and needs to be modeled in a cinematic way. Moreover, the image motion speed in the field of view is measured by its angular velocity relative to the camera coordinate system under the single-degree-of-freedom eye-neck motion mechanism. It is a linear relationship, corresponding to the bionic eye mechanism, whose motion speed should be measured by the movement speed of the target point imaging in the pixel coordinate system, which is nonlinear.

### 3.2 Wide-field Bionic Compound Eye Target Recognition and Detection Algorithm

#### 3.2.1 Wide Field of View Bionic Compound Eye Target Recognition Stud

**FIGURE 2:**
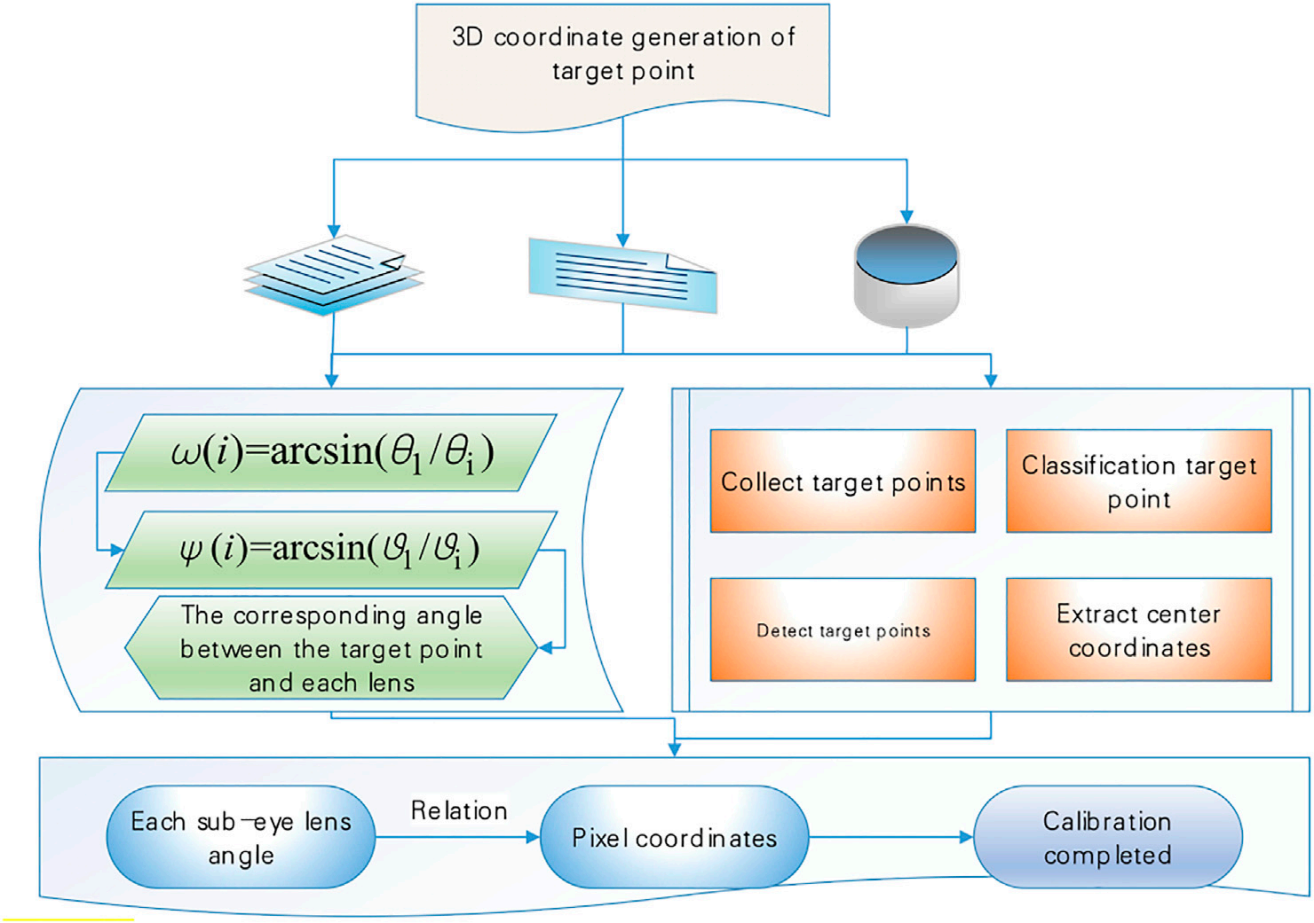
Calibration of Curved Variable Focal Length compound eye imaging system.

To localize the target point in three dimensions, the first part of the target localization model of the compound eye imaging system can be solved by linear calculation. Based on the machined microlens array mold, it is known that the center of each sub-eye lens of this curved variable focal length compound eye model has been fixed, and a set of super qualitative Equations can be determined based on the multiple sub-eye lenses that capture the target point to solve for the target point 3D coordinates. The calibration task is to find out the relationship between the incident light vector angle of each sub-eye lens and its corresponding target image point, to pave the way for finding the corresponding incident light vector angle from the captured target image point. The calibration flowchart of the curved variable focal length compound eye imaging system is shown in Figure 2.

If the overall calibration of the compound eye imaging system is to be established, the relationship between the vector angle of the incident light direction of a single sub-eye lens and its corresponding image point should be established firstly, which can be derived from a known three-dimensional target, as in .

$$\{\begin{array}{c} \omega \left(i\right)=\theta_{i}*\frac{A-A\left(O_{i}\right)}{C-C\left(O_{i}\right)}\\ \psi \left(i\right)=\vartheta_{i}*\frac{B-B\left(O_{i}\right)}{C-C\left(O_{i}\right)} \end{array}$$ (6)

The direction vector formed by the target point and each sub-ocular lens can be found separately.

$$\overset{\Rightarrow }{H}=\left(0,\frac{\omega \left(i\right)}{\zeta \left(i\right)},\frac{\psi \left(i\right)}{\zeta \left(i\right)},1\right)$$ (7)

The vector incidence angle corresponding to each sub-eye lens can be obtained, as in Eq. 8. When there are enough image points, to cover the entire image plane of the image detector, the correspondence between the vector angle of all incident rays within the field of view of the sub-eye lens and the image points can be obtained by .

$$\{\begin{array}{c} \omega \left(i\right)=\text{arcsin}\left(\theta_{1}/\theta_{\text{i}}\right)\\ \psi \left(i\right)=\text{arcsin}\left(\vartheta_{1}/\vartheta_{\text{i}}\right) \end{array}$$ (8)

When a spatial target is captured by a sub-eye of the compound eye, the mapping relationship between the established target angle and the spot coordinates can be used to reverse the mapping to obtain the angle value of the spot on the two virtual double spherical rake markers, knowing the angle information can calculate its three-dimensional spatial coordinates $A\left(a_{1},b_{1},c_{1}\right),B\left(a_{2},b_{2},c_{2}\right)$ , and the straight line determined by the two points is the incident light line, as shown in .

$$F\left(a,b,c\right)=\frac{a-a_{1}}{a_{2}-a_{1}}=\frac{b-b_{1}}{b_{2}-b_{1}}=\frac{c-c_{1}}{c_{2}-c_{1}}$$ (9)

For the verification set of spot images, we first need to extract the center coordinates of the spot and match them to the corresponding sub-eye channels and then use kriging interpolation to obtain the corresponding angle information based on the established nonlinear mapping relationship between the target point angle and the spot coordinates under each sub-eye. Suppose the actual position of the target point is [a(i),b(i),c(i)] , and the coordinates of the target point obtained by using the calibrated compound eye are $\left[a{\left(i\right)}^{'},b{\left(i\right)}^{'},c{\left(i\right)}^{'}\right]$ , the positioning error can be expressed by the lateral angle error and the overall angle error, and the overall angle error is shown in .

$$\forall \rho =\frac{\sqrt{{\left(a\left(i\right)-a{\left(i\right)}^{'}\right)}^{2}+{\left(b\left(i\right)-b{\left(i\right)}^{'}\right)}^{2}+{\left(c\left(i\right)-c{\left(i\right)}^{'}\right)}^{2}}}{\sqrt{a{\left(i\right)}^{2}+b{\left(i\right)}^{2}+c{\left(i\right)}^{2}}}*100\%$$ (10)

In the cloud service network, each grid will detect multiple range frames. However, during the training process of the network, it is desirable to have only one target frame to participate in the detection for each detection target. Therefore, the cloud service network will select the range frame with the largest IoU value to detect the target, so that each object will have a suitable and specific range frame to be detected. When the object in the image is large or the object is at the boundary of the grid, more than one grid may be localized, and the cloud service network uses non-maximum suppression to remove duplicate detected objects.

#### 3.2.2 Wide Field of View Bionic Compound Eye Target Detection Study

First, the whole image is segmented with a positive hexagonal profile, and the side lengths of the hexagon need to be appropriately selected according to the image resolution, target size, etc. There are two options for the interpretation of the segmentation results. When the target of analysis is the motion information between adjacent small eyes, each hexagon as a small eye. Option two, treat each hexagon as the center of one small eye, and the six neighboring hexagons around it as the photoreceptors in the same small eye. If the second scheme is adopted, we have to reduce the size of the hexagon appropriately to improve the spatial resolution. Both are similar in the final processing method and results, and the second scheme is adopted in this paper. Each eyelet contains seven hexagons, and there is an overlap of hexagons between two adjacent eyelets in the horizontal direction, and no overlap in the vertical direction.

To improve the spatial resolution of the motion detection, a hexagon with a side length of three pixels is used in the practical implementation. For the input raw image, it is first converted into a grayscale image. Next, the grayscale image is Gaussian smoothed to reduce the effect of random noise, and a standard Gaussian template of 4 × 4 is used here. Then, the luminance of all pixels in each hexagon is averaged as the luminance value of the hexagon. N(x) is the number of pixels in the hexagon, Q (i,j) is the luminance of the pixels in the hexagon; P (i,j) represents each pixel in the hexagon. To low-pass filter the luminance signal of each hexagon in the time domain, it is necessary to buffer a sufficient number of frames (the default value in this paper is N_Frame = 32 frames.

$$H\left(x\right)=\frac{\sum_{i,j=0}^{6}Q\left(i,j\right)*P\left(i,j\right)}{N\left(x\right)}$$ (11)

**FIGURE 3:**
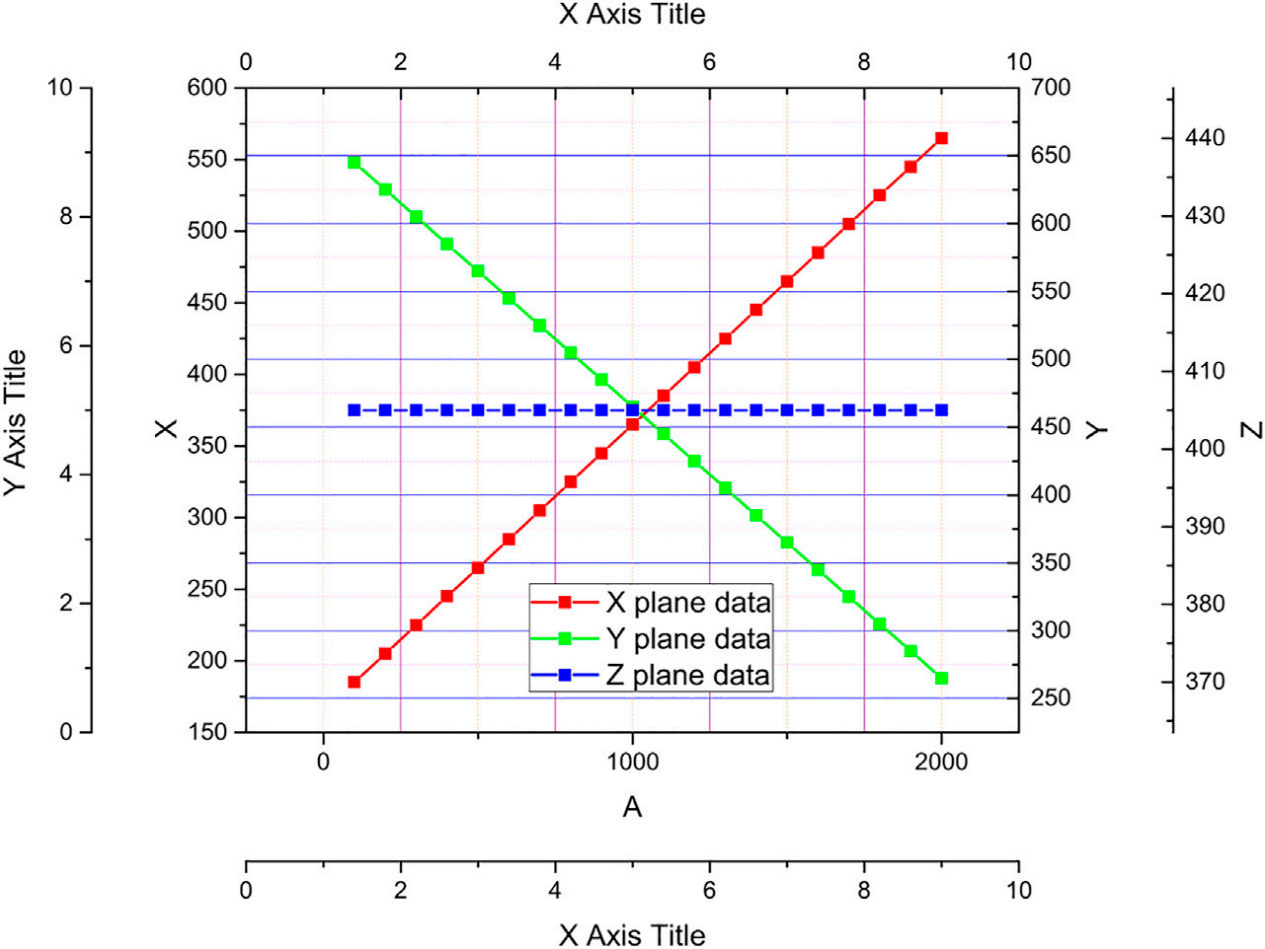
Fading point to determine the initial point of the target plane schematic.

Define the target initial point as the intersection of the optical axis and the target plane, and use this as the center to solve the three-dimensional coordinates of the target point, the compound eye localization test system established according to the fading point principle overlaps the four light spot image planes collected by constantly changing the distance between the target plane and the light source, thus determining the initial point of the target plane as shown in Figure 3, and the pixel coordinates of the initial point of the target plane can be known as (1,000, 375). With 50 light spots collected, the 3D coordinates of the target point can be calculated by the target localization model established above. The arrangement of the sub-eyes is optimized, 251 sub-eyes are arranged on the bionic spherical compound eye by the method of icosahedral subdivision, and the original plano-convex lens is replaced with a logarithmic axicon with a better imaging effect.

It can be seen from the experiments of the surface variable focal length compound eye target point position test system that after the test system is tuned, the coordinates of each light source point in the target plane with the three-dimensional world coordinate origin (the center of the compound eye central sub-eye lens optical axis center) as the coordinate system can be found according to Eq. 12. where a 1) and b(1) are the coordinates of the pixel coordinate system in the target plane for any point, respectively; c(i)_0_ is the initial distance of the target sought; a, b, c are the three-dimensional coordinates of the target point in the world coordinate system.

$$\{\begin{array}{c} a=\left(a\left(i\right)-a{\left(i\right)}_{0}\right)*0.438\\ b=\left(b\left(i\right)-b{\left(i\right)}_{0}\right)*0.318\\ c=c{\left(i\right)}_{0}*0.143 \end{array}$$ (12)

The system state x is defined as the motion state of the neck and the eye, the system input I(e) is the angular acceleration of these two respective joints, and the system output y is some states of interest including feature point imaging angle, which can be written as .

$$\{\begin{array}{c} a=\left(\vartheta \left(e\right),\theta \left(n\right),\psi \left(e\right),\psi \left(n\right)\right)\\ b=\left(\vartheta \left(e\right),\vartheta \left(n\right),\theta \left(e\right),\theta \left(n\right),\psi \left(e\right),\psi \left(n\right)\right)\\ I\left(e\right)=\left(\alpha \left(e\right),\beta \left(n\right)\right) \end{array}$$ (13)

Motion controllers are designed to follow a certain metric, which makes tracking the best possible. By analogy with the performance of the human visual system during sweeping motion, it is not difficult to find some evaluation functions of the human visual system during motion. First, the evaluation function is shown in Eq. 14, the matrix P is the terminal performance metric by structure.

$$M\left(P\right)=\frac{\sum_{i=1}^{n}e*I{\left(f\right)}^{N}*P\left(e\right)\left(I_{i}^{f}\right)}{3}$$ (14)

For to track the target under the most tracking performance index, an attempt is made to find the optimal control rate using the Hamiltonian Equation. The optimal control I(t) is obtained as shown in .

$$I\left(t\right)=\frac{M\left(t\right)*a\left(t\right)-b\left(t\right)}{-M^{-T}*P^{T}}$$ (15)

### 3.3 Design and Analysis of Wide-field Bionic Compound-Eye Target Recognition and Detection System

After specifying the index parameters of the system and theoretically analyzing the possible main aberrations of the system, ZEMAX is used to design the system, and the actual aberrations in the system are analyzed and evaluated in real-time, and the corresponding methods are used to balance the aberrations and optimize the image quality to improve the system performance. After system optimization, the system is assigned tolerances and its processing performance is analyzed after all index parameters of the cloud service network. Bionic compound eye system are met and the image quality performance exceeds the target requirements. The initial structural parameters of each sub-eye in the microlens array were calculated by using the spaced surface bionic compound eye parameter calculation model, as shown in Table 2.

**TABLE 2:** Sub-eye initial structure parameters.

Surface Type	Radius of curvature (um)	Thickness (um)	Material
X surface	314	435	PMMA
Y surface	259	355	PMMA + QMA
Z surface	138	265	PMA + GMA

The aberration analysis charts, such as the optical path diagram, light fan diagram, and point column diagram of the initial structure of the sub-eye system, are used to analyze the aberration of the system in the on-axis field of view. The light rays hitting the detector after ray tracing do not converge at the same point completely, but show a diffuse distribution; the light rays at different angles to the optical axis have different deviations from the ideal image point after ray tracing through the lens.

After converting the technical index requirements of the cloud service network bionic compound eye system into the cloud service network parameters required for system design, it is necessary to find the matching initial structure from the existing literature or patent database as the starting point of the design, and this initial structure needs to have as many target parameters as possible to meet the required cloud service network parameters. Usually, the suitability of the initial structure selection determines the subsequent efficiency of the cloud service network designer to a certain extent. Cloud service network design is an iterative process of solving multivariate equations. If the initial structure is chosen to be easier to approach the demand solution as the design starting point will bring convenience to the system optimization. Otherwise, it may make the design inefficient and even difficult to complete the design requirements, therefore, the initial structure of the bionic compound eye system of the cloud service network is selected appropriately or not to the final design result is crucial. Based on the previous design experience and the field of view parameters of the system to be designed, a cloud service network bionic compound eye system with the required field of view is built as the initial structure of the trans-imaging system. In this paper, a monitoring system with a fixed camera and PTZ camera is implemented and applied to the actual monitoring scene. They statistically analyze the performance of the system through the experimental results, and the results show that the system is a relatively successful and reasonable intelligent monitoring system.

**FIGURE 4:**
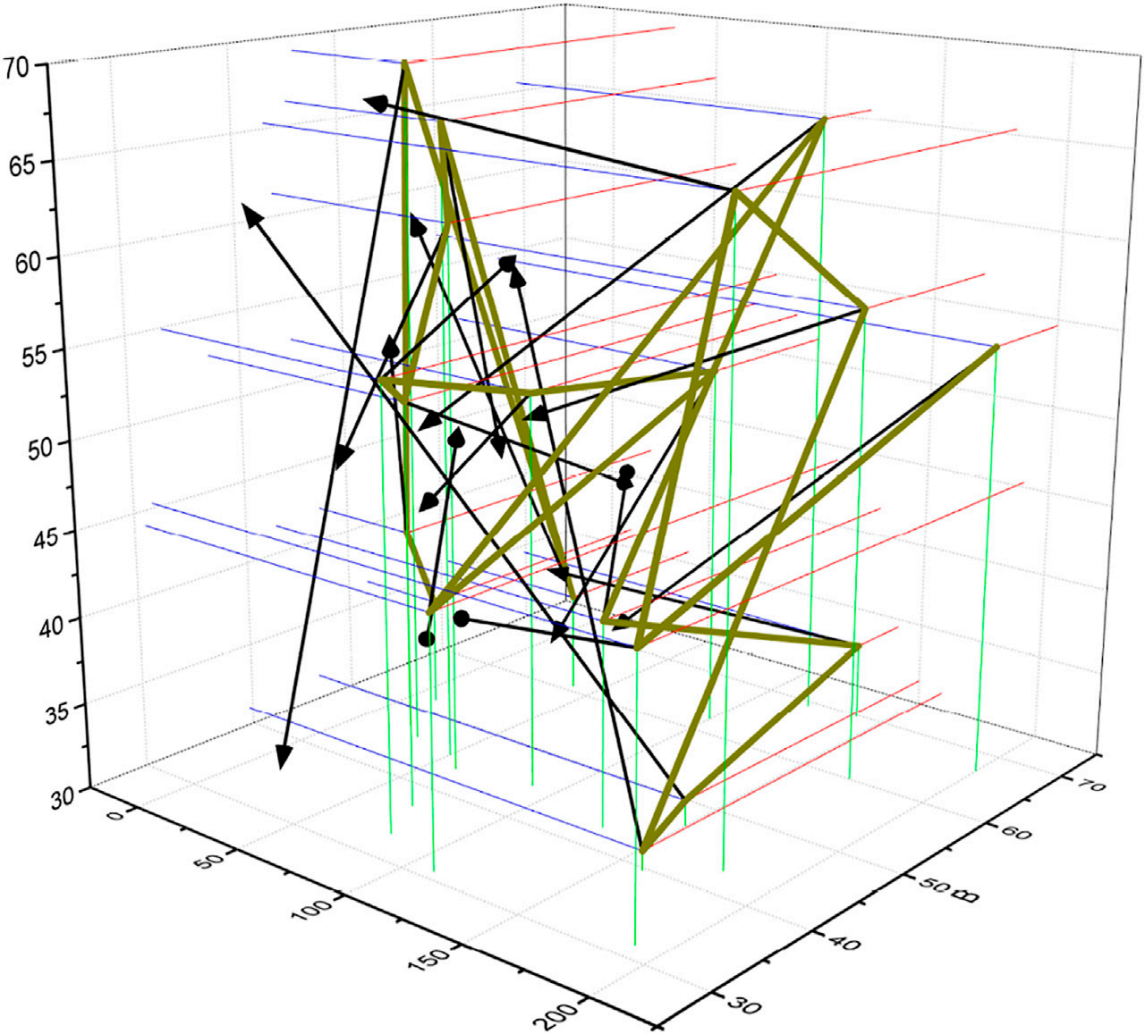
Initial structure point column diagram.

After finding a structure close to the required target parameters from the literature, the focal length is scaled, and then the field of view, aperture, and other related parameters are gradually changed so that the parameter index of the structure can meet the requirements of the required cloud service network bionic compound eye system. Since the modification of the parameters and materials in the cloud service network bionic compound eye system will introduce a large number of aberrations into the system, it is necessary to use various effective methods to analyze the aberrations and optimize the system again to obtain a cloud service network structure with good image quality that meets the required cloud service network specifications. Figure 4 shows the point column plots reflecting the imaging performance of the initial structure after ray tracing. The RMS root means the square radius of the initial structure is larger than the size of a single pixel of the selected detector, which cannot meet the required imaging requirements, and there is a comet-like trailing phenomenon in the dot plot of the edge field of view, and the dot plot of each wavelength is also There is no overlap, that is, aberrations such as coma and chromatic aberration of the system need to be further optimized.

The radius of curvature of the object plane is set to be equal to the radius of curvature of the focal surface image formed by the microlens array. The initial structure of the transcendental image system is simulated and analyzed by using ZEMAX, and the aberration correction is performed by controlling the lens parameters, material matching, etc. The evaluation function is used to optimize the cloud service network bionic compound eye system. The evaluation function is used in the cloud service network design software to define the performance merit of the cloud service network bionic compound eye system, which has an ideal value of 0. Each operand in the evaluation function represents the target to be evaluated, the actual value of the current cloud service network bionic contained compound eye system and the set target value. The error caused by each operand is shown in Eq. 16, where f(10) represents the current actual value and t(10) represents the desired target value.

$$\psi \left(x\right)=\sum_{i=1}^{N}f\left(x\right)-t\left(x\right)$$ (16)

## 4 Analysis of Results

### 4.1 Cloud Service Network Model Analysis

**FIGURE 5:**
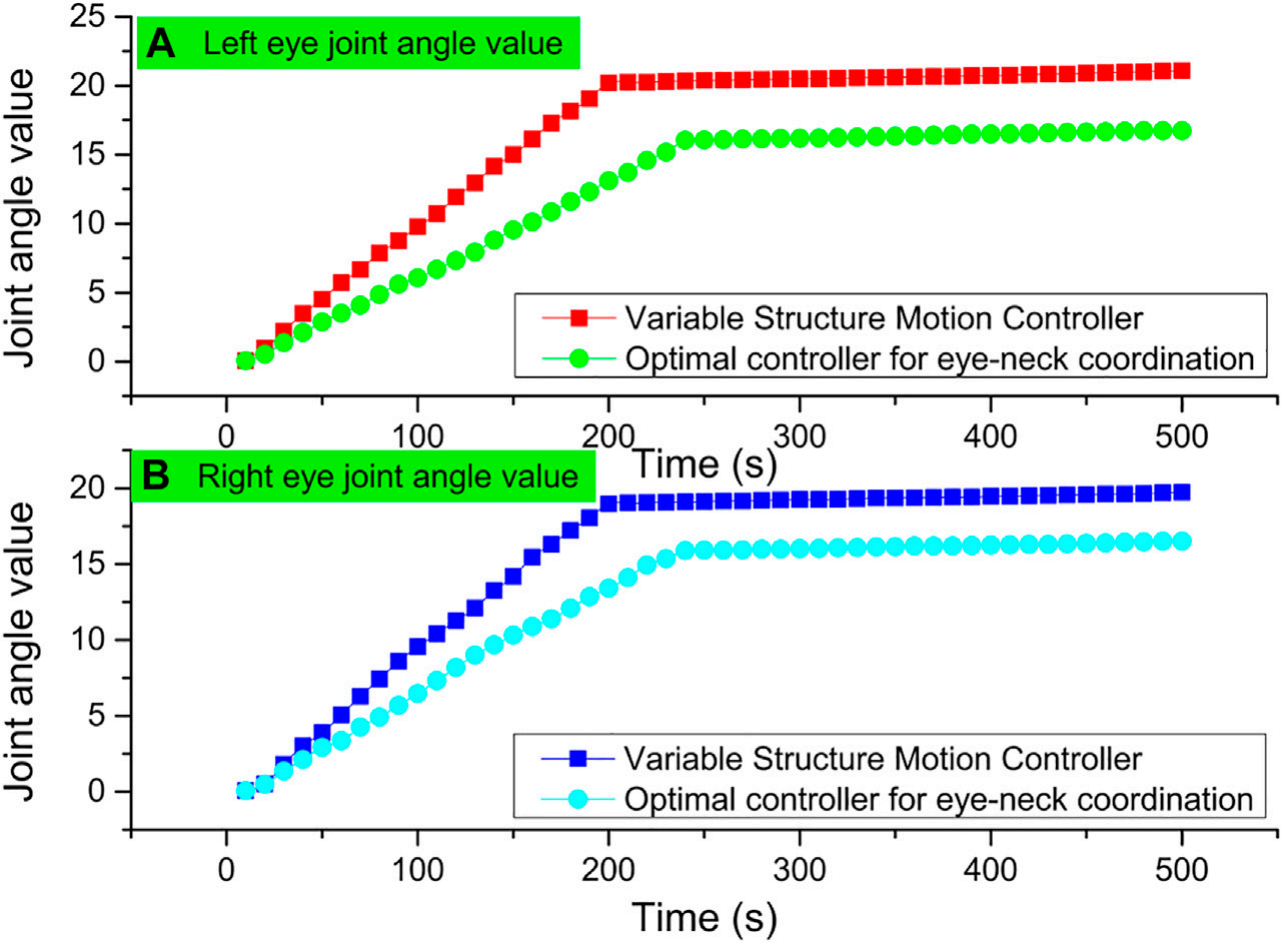
Variable structure controlled stationary target tracking data.

A sphere is placed laterally in front of the bionic eye, and using the designed control scheme, the starting position of the bionic eye is set to 0° for all joint angles, which puts the bionic eye in a horizontal forward-looking action; the tracking target is set to a stationary sphere using the feature point and optical flow-based image tracking algorithm; the bionic eye is controlled by the eye-neck coordinated optimal controller and the variable structure motion controller, respectively. The field of view of the bionic eye is moved toward the center of the sphere using the eye-neck coordinated optimal controller and the variable structure motion controller, respectively. As seen from the experimental process, both of them achieve the tracking of the stationary target by controlling the coordinated motion of the bionic eye and the neck. The joint angle data of both during the tracking motion are shown in Figure 5. As seen in Figure 5, the variable structure controls the stationary target tracking data to have a stable value over time, and the value is 21.02. The eye motion and neck motion controlled by the variable structure controller is more like moving at the same pace, which is only a simple sharing of the target tracking motion task and lacks the advantage of complementing each other.

**FIGURE 6:**
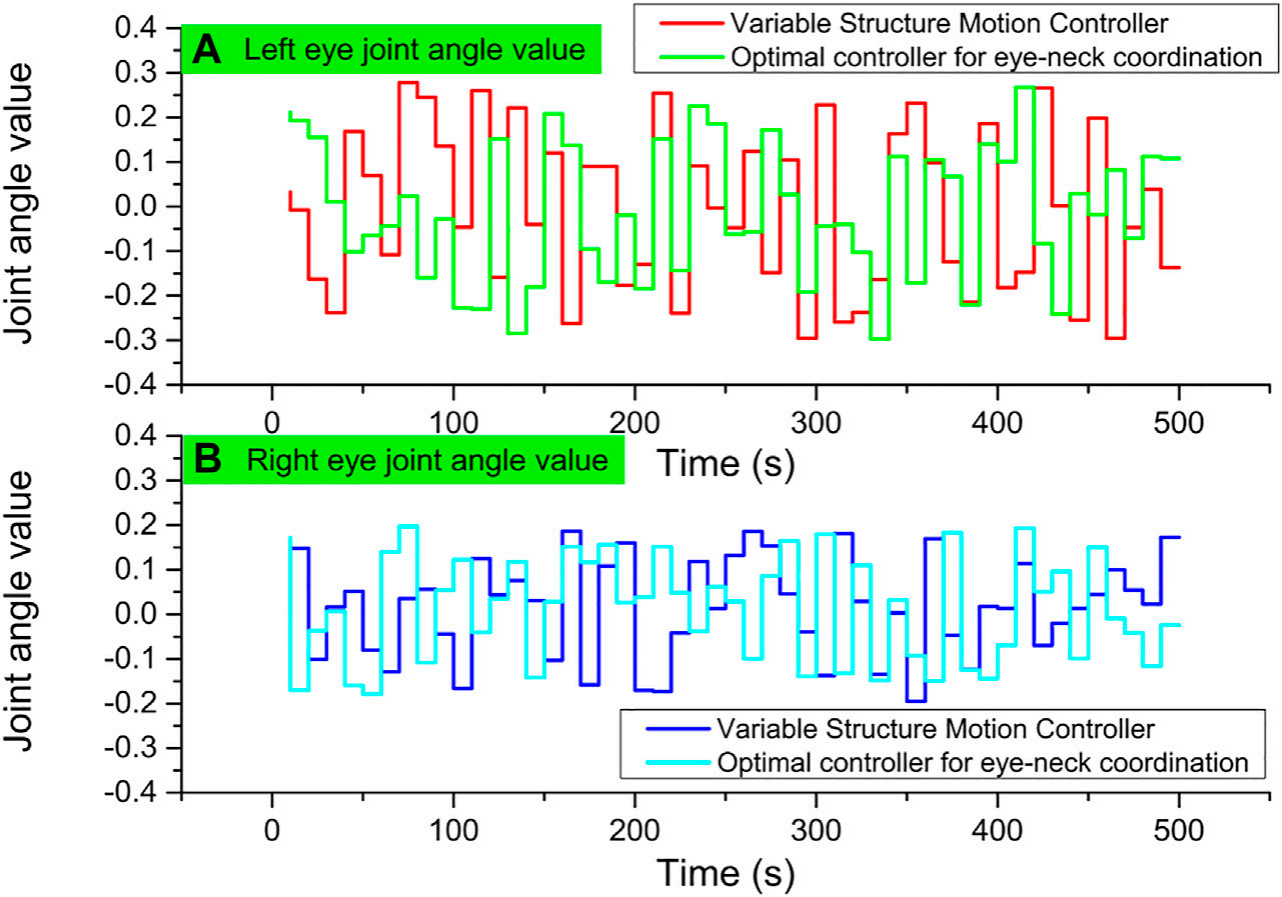
Optimal control of human-dragged target tracking data.

The target tracking process uses two schemes, the eye-neck coordinated optimal controller and the variable structure controller, to track similar human dragging movements, respectively. Among them, the joint angle data obtained during the target tracking process using the eye-neck coordinated optimal controller are shown in Figure 6.

The tracking effect of the eye-neck coordinated motion controller is also smoother and better than that of the variable structure controller in pitch joints that do not reach the ultimate position. The unsmooth tracking of the variable structure controller will produce back and forth switching under certain circumstances, which leads to a decrease in smoothness, and is not conducive to image processing. The complementary advantages of eye and neck movements in eye-neck coordinated motion are still an important reason for its more excellent tracking effect. Finally, the motion of the bionic eye controlled by the eye-neck coordinated optimal controller is very similar to the tracking motion of the human visual system, and the human-computer interaction is better.

### 4.2 Algorithm Performance Analysis

**FIGURE 7:**
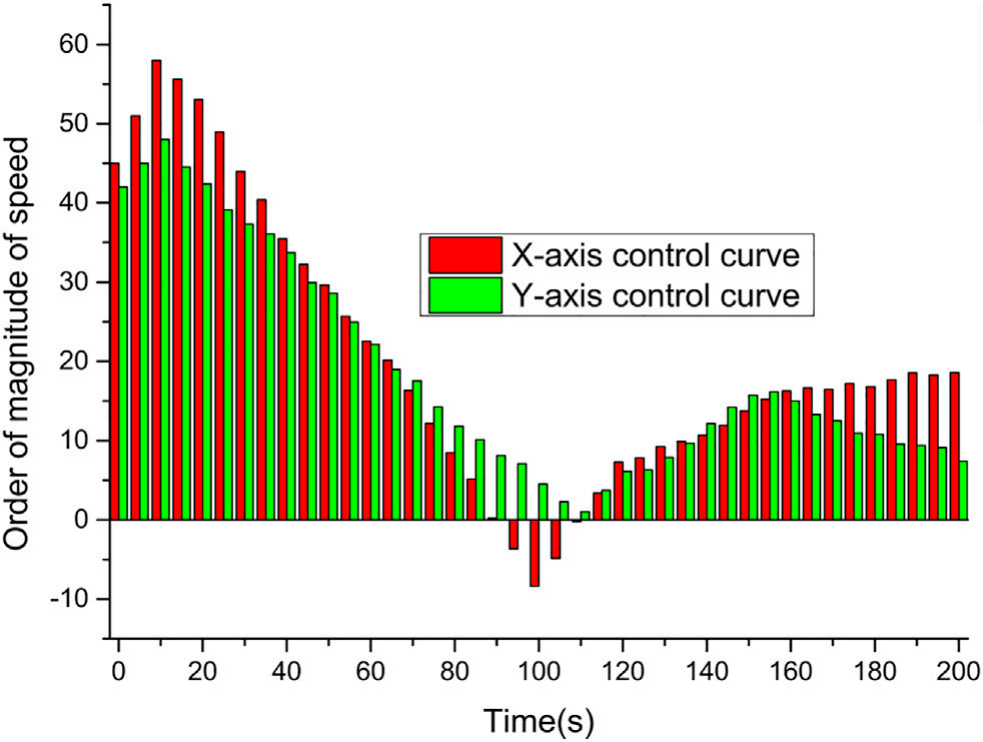
The effect of uniform motion target tracking.

The tracking effect of the uniform motion target of the cloud service network after velocity loop correction detection control is shown in Figure 7. As can be seen in the tracking curve of the uniform motion target in Figure 7, after introducing the velocity step response model of the ball machine and using the detection control algorithm for velocity loop correction, compared with the state feedback method based on the first-order inertial link model, the impact of the model mismatch on the control performance is greatly reduced, and the tracking curves of both horizontal and vertical axes are smoother, while the control jitter amplitude can still be limited. After comparison, it can be found that the velocity loop correction method based on detection control can not only achieve better tracking performance of the moving target, but also meet the control requirements of the smooth control volume. It is effective in terms of both control performance and actuator protection.

**FIGURE 8:**
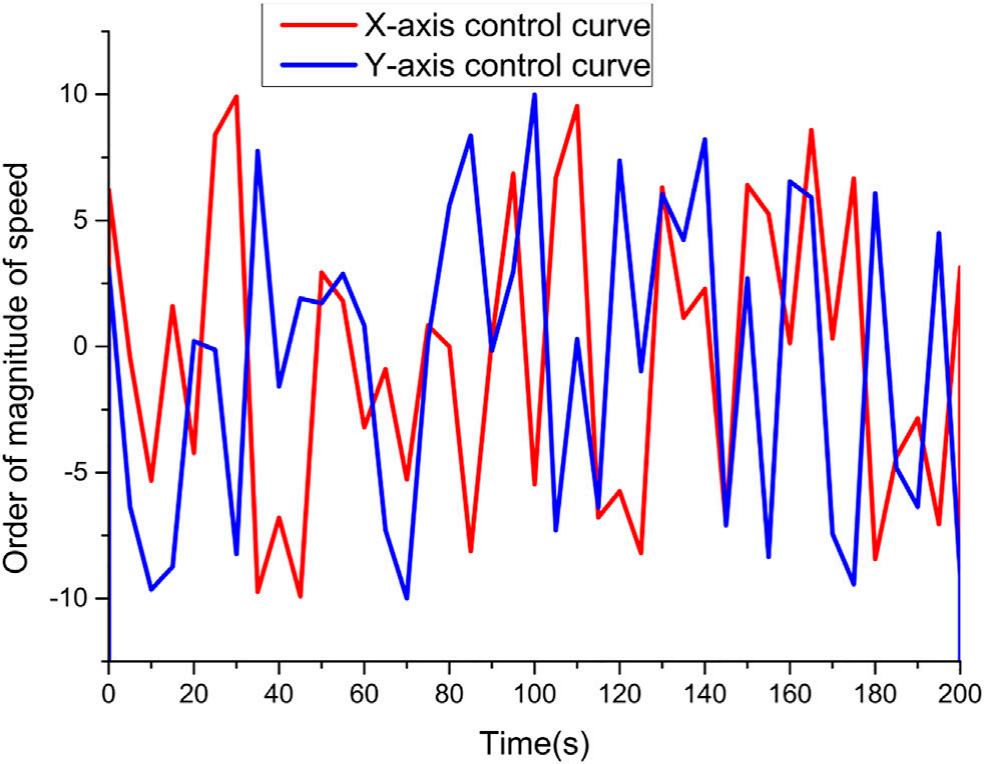
Following target tracking effect.

The tracking effect of the detection control combined with the head and shoulder detector on the following target is shown in Figure 8. From Figure 8, it can be seen that the controller tracking performance is better when the pedestrian moves smoothly in one direction. However, when the target suddenly turns back, there is a certain degree of overshoot in the ball machine tracking, and when the target speed is larger, the overshoot phenomenon will be more serious, which is caused by the strong maneuverability of the moving target. The speed regulation inertia of the actuator and other reasons together and is also a key problem in the tracking control of the following target.

**FIGURE 9:**
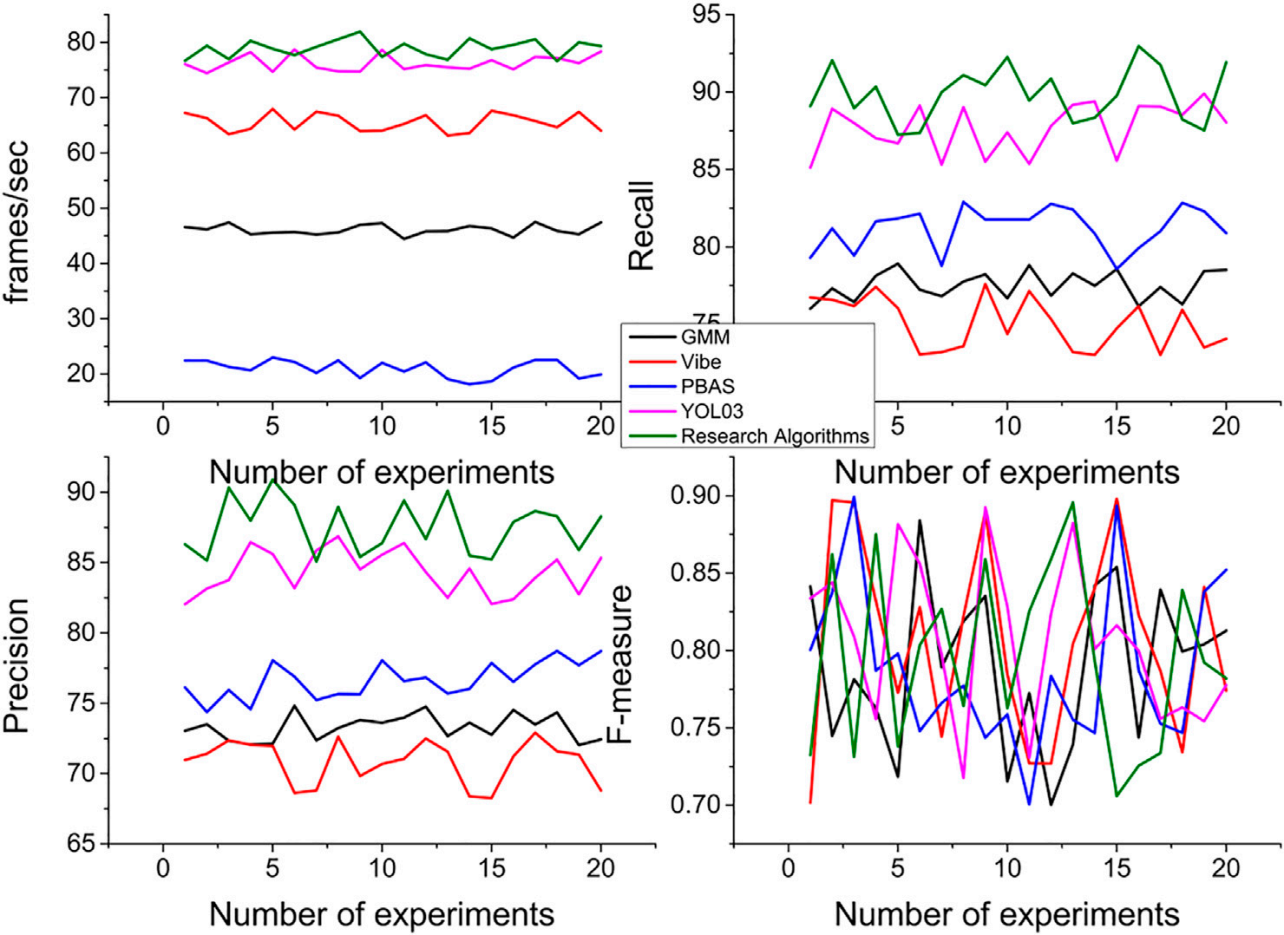
Average performance comparison of algorithms.

Figure 9 compares the time, recall, precision, and F-measure of the algorithms by counting the processing results of the various methods on these videos.

From the experimental results, we can see that the algorithm in this paper performs average in the recall rate, better in the precision, and the F-measure is more desirable. The missed detection is mainly caused by the large threshold of motion information, and if the threshold is lowered, the missed detection rate decreases and the false detection rate increases, and the two need to be handled in a compromise. In terms of processing speed, it is better than GMM, Vibe, and PBAS methods, and weaker than the YOLO3 algorithm. In terms of qualitative indicators, the algorithm in this paper has low overall operational complexity due to its motion-triggered nature, as well as low false detection rate; and the local motion detector used is insensitive to illumination changes and randomness jitter, and thus can better handle scenarios such as illumination changes and camera jitter. However, this method has low foreground target accuracy and ineffective detection of too-small targets compared with the background modeling-based method, the threshold needs to be dynamically adjusted to effectively detect targets with different motion speeds, and non-continuous motion target detection needs to be supplemented with motion tracking.

### 4.3 System Simulation Analysis

**FIGURE 10:**
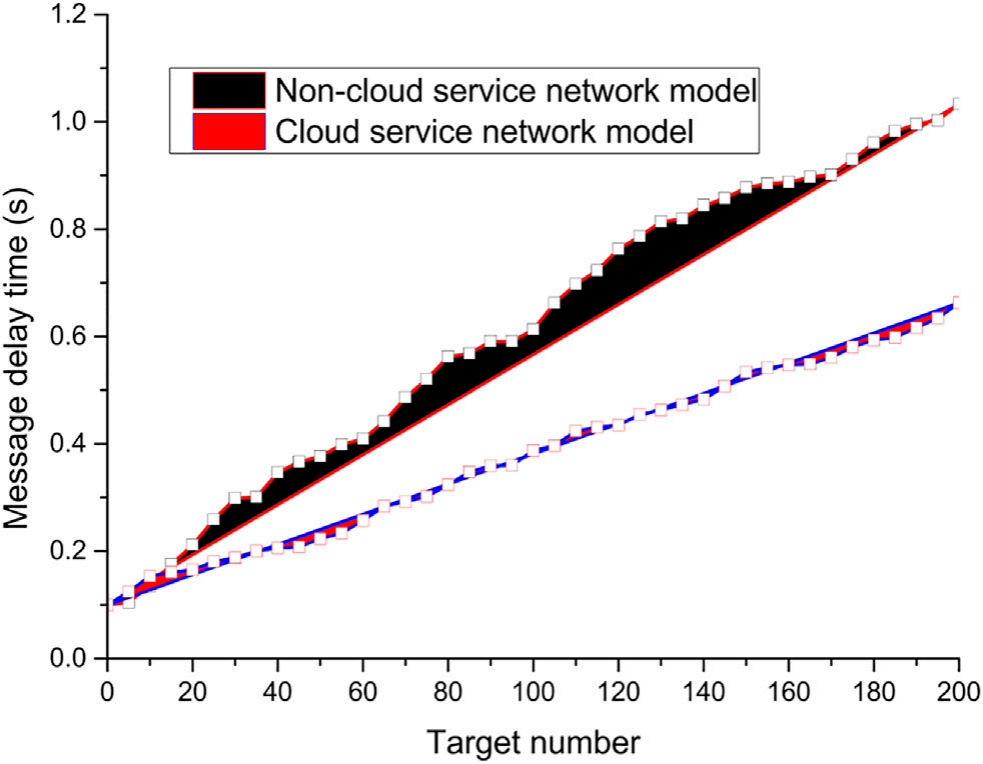
Message delay analysis and comparison.

In the experimental results, the analysis and comparison for the message latency are shown in Figure 10, where the message latency increases with the increase in the number of targets. It can be seen clearly from Figure 10 that the value of the message latency parameter of the case model constructed using the proposed cloud service network is significantly lower than that of the scheme model without the cloud service network. As the number of targets increases, the message delay also increases, but the message delay increases more gently in the case model using the cloud service network, while the message delay increases significantly more in the experimental model without the cloud service network, and the parameter value of the message delay is significantly larger than that of the experimental model using the cloud service network. It can be seen that using the cloud service network model scheme proposed in the paper can effectively reduce the message delay of the cloud service network, which ensure the timely transmission of the target messages.

**FIGURE 11:**
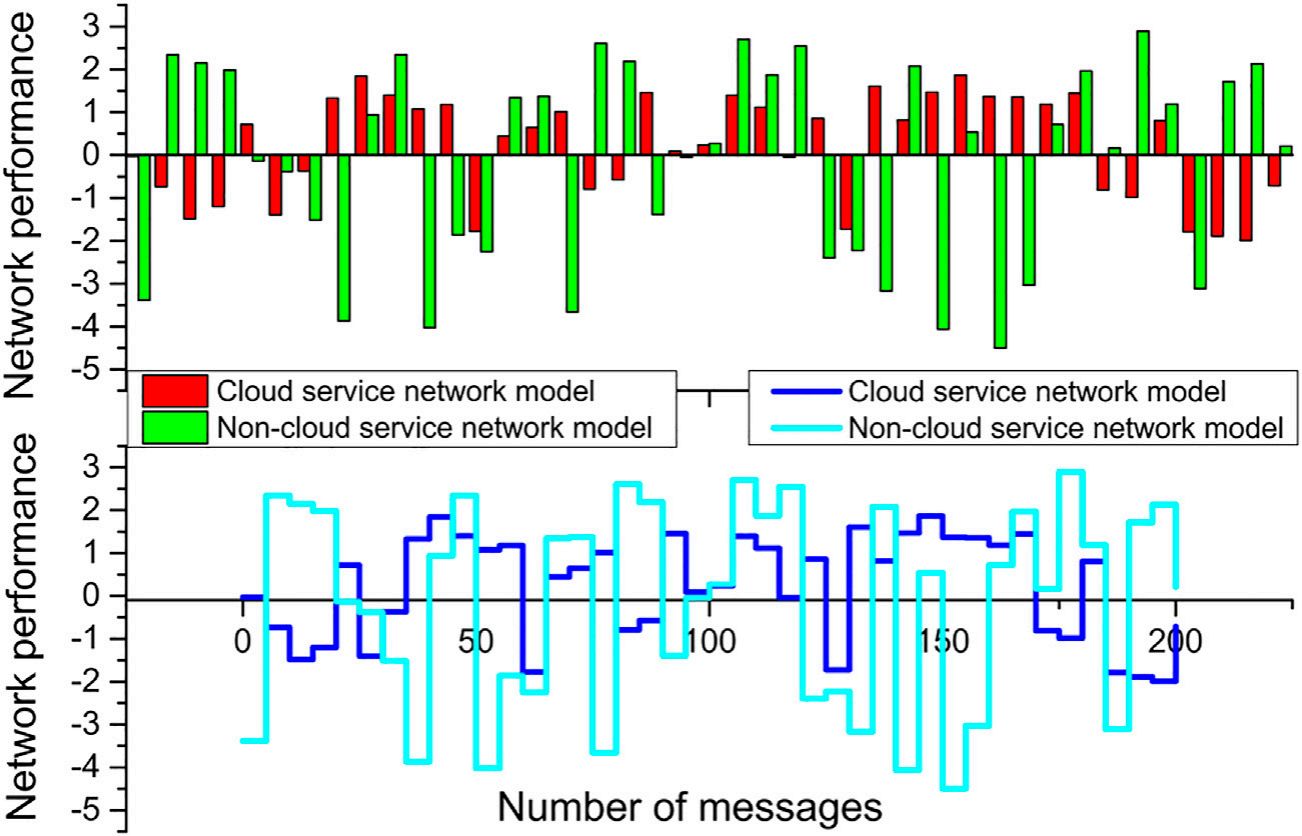
Network jitter analysis and comparison.

As shown in Figure 11, the value of the network jitter parameter fluctuates up and down in a certain range with the number of messages transmitted by the nodes. And from Figure 11, it can be found that with the increase in the number of messages transmitted by the target, the network jitter value fluctuates between (−2, 2) in the model scenario where the cloud service network is applied, with a smaller range of up and down fluctuation; while in another model where the cloud service network is not applied, the network jitter value fluctuates up and down more obviously with the increase in the number of target messages, with its jitter value fluctuating between (The jitter value fluctuates between (−3, 5), which indicates that the performance jitter of each node in the network changes more obviously, and its overall network performance is more unstable.

**FIGURE 12:**
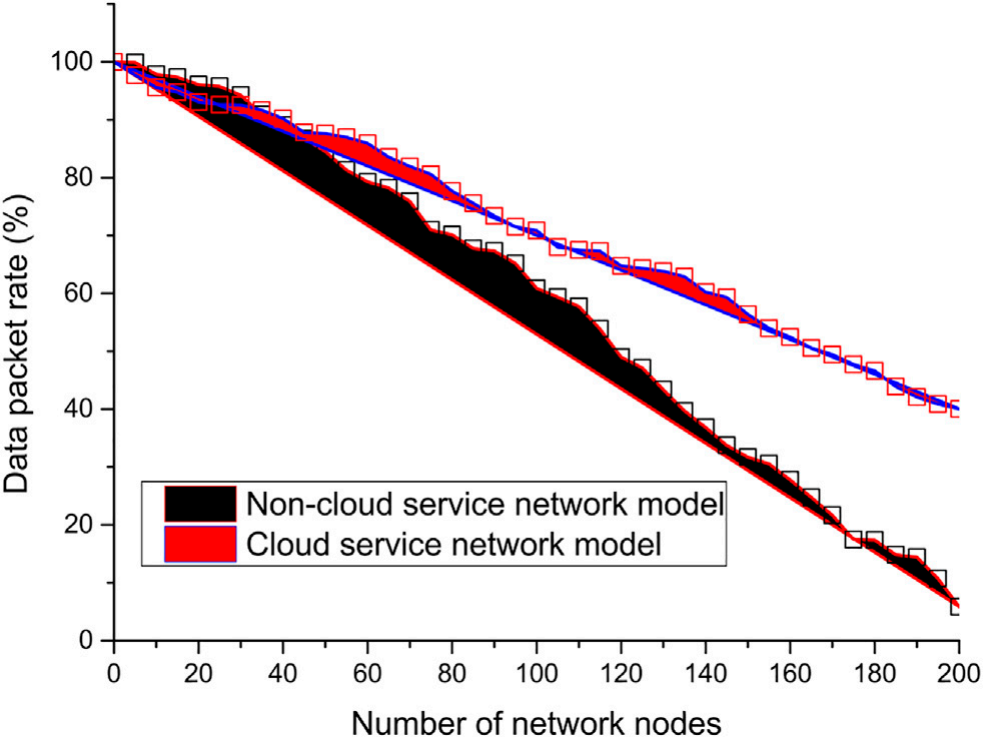
Analysis and comparison of packet delivery rates.

The article analyzes and compares the variation of system overhead with packet delivery rate, as shown in Figure 12. The packet delivery rate refers to the percentage of packets that are successfully received when they are sent or transmitted from the source node to the destination node, and the higher the packet delivery rate, the higher the reliability of the network. Overall in the cloud service network architecture, the increase in the number of network nodes will cause the network performance to be affected in some way, such as the increase in network overhead and the decrease in packet delivery rate. As shown in Figure 12, as the number of onboard nodes increases, the packet delivery rate tends decrease. However, the packet delivery rate of the model with a cloud service network is decreasing, but the magnitude is significantly lower than that of the architecture model without a cloud service network; in the architecture model without a cloud service network, the packet delivery rate is decreasing sharply. The cloud service network architecture model has a better packet delivery rate and also proves the effectiveness of the proposed architecture.

## 5 Conclusion

This paper study the wide-field active vision tracking system, which is composed of PC, fixed cameras, and cloud service network, and is capable of the wide field of view motion target detection and high-resolution image storage. The video sequence of the fixed camera uses a detection algorithm to get the pedestrian position, the control platform as the processing center completed the video acquisition and display of dual cameras, serial transmission, human-computer interaction, etc. The innovation and application of network technology and communication technology have greatly promoted the development of cloud service networks, which make the architecture system, communication mode and user experience of cloud service networks to be continuously innovated. The wide promotion and application of cloud service network promoted the interaction and sharing of traffic service information and entertainment service information, and improving the wide-field bionic compound-eye target recognition and detection technology. The stability and speed of the wide-field bionic compound-eye target recognition and detection algorithm is an important factor in evaluating the performance of the whole system, and also a difficult point of development. The conventional recognition and detection algorithm is easy to understand and simple to implement, and the tracking effect is not good due to the inertia of the cloud service network itself as well as the delay of instruction processing. In response to this phenomenon, other measures are taken to optimize and improve, successively combining state space and detection control for control, which improves the stability and speed of the system. Since the algorithm in this paper takes into account the objective evaluation parameters that provide the effect of the exhibition, the disturbance factors are relatively few (Jiang et al., 2021a; Jiang et al., 2021b).

## Data Availability

The original contributions presented in the study are included in the article/Supplementary Material, further inquiries can be directed to the corresponding author.
